# Sarcopenia in Thai community-dwelling older adults: a national, cross-sectional, epidemiological study of prevalence and risk factors

**DOI:** 10.1186/s12889-024-17804-7

**Published:** 2024-01-27

**Authors:** Ekasame Vanitcharoenkul, Aasis Unnanuntana, Pojchong Chotiyarnwong, Panai Laohaprasitiporn, Nath Adulkasem, Apichat Asavamongkolkul, Chandhanarat Chandhanayingyong

**Affiliations:** Department of Orthopaedic Surgery, Faculty of Medicine Siriraj Hospital, Mahidol University, 2 Wanglang Road, Bangkok Noi, Bangkok, 10700 Thailand

**Keywords:** Elderly, National epidemiological survey, Prevalence, Risk factor, Sarcopenia

## Abstract

**Background:**

Sarcopenia is an age-related condition characterized by a progressive loss of skeletal muscle mass. It leads to declining physical performance, potentially culminating in a diminished quality of life or death. This study investigated the prevalence of sarcopenia and its associated risk factors among Thai community-dwelling individuals of advanced age.

**Methods:**

Between March 2021 and August 2022, we conducted a nationwide community-based epidemiological survey across all six major regions of Thailand. Participants with sarcopenia were identified according to the 2019 criteria of the Asian Working Group for Sarcopenia (AWGS). The risk factors were examined using multivariable logistic regression.

**Results:**

Of the 2456 participants, the overall prevalence of sarcopenia was 18.1%, with nearly two-thirds (66.9%) classified as having severe sarcopenia. Multivariate analysis identified six associated risk factors for sarcopenia. They are a lower body mass index (odds ratio [OR] = 11.7, 95% confidence interval [CI] = 7.8–17.4), suboptimal leg calf circumference (OR = 6.3, 95% CI = 4.3–9.5), male sex (OR = 2.8, 95% CI = 2.2–3.7), a history of chronic obstructive pulmonary disease (OR = 2.3, 95% CI = 2.3–5.0), advanced age (OR = 2.1, 95% CI = 1.3–3.3), and an increasing time in the timed up-and-go test (OR = 1.1, 95% CI = 1.0–1.1).

**Conclusions:**

This is the first large-scale national study to represent the prevalence and risk factors for sarcopenia in Thai community-dwelling individuals of advanced age using the AWGS 2019 criteria. Interventions such as lifestyle modifications and appropriate nutrition should be promoted throughout adulthood to maintain muscle strength and delay the onset of sarcopenia, particularly in males.

**Trial registration:**

The Central Research Ethics Committee of the National Research Council of Thailand authorized the study protocol (approval number COA-CREC023/2021).

**Supplementary Information:**

The online version contains supplementary material available at 10.1186/s12889-024-17804-7.

## Background

The global population of older individuals has witnessed significant growth in recent years. In 2021, Thailand joined Singapore as the second ASEAN country to be classified as an “aged society,” with the Thai population aged 60 and over accounting for 20% of the total population. Projections suggest that this figure will escalate to 30% by 2035, pushing Thailand into the “superaged society” bracket [1]. This swift demographic transition signals a probable uptick in age-related diseases among these older groups. One of the most prevalent age-associated conditions is sarcopenia, marked by a steady decline in skeletal muscle mass and physical strength, culminating in either a diminished quality of life or fatality [2]. Despite growing awareness around diagnosing sarcopenia, early detection and prevention methodologies remain inadequate.

A disparity in sarcopenia prevalence across the globe can be attributed to a mixture of diagnostic criteria and the heterogeneity of target populations. Research conducted in 2021 through a systematic review and meta-analysis revealed sarcopenia prevalence rates ranging from 10 to 27%, with prevalence tending to increase with the age of the population [3]. Ethnicity and the environment substantially influenced these rates. Accordingly, several diagnostic criteria have been developed specifically for each population. In 2014, the Asian Working Group for Sarcopenia (AWGS) developed criteria and cutoff values for diagnosing sarcopenia in the Asian population [4]. These criteria were updated in 2019 [5].

The reported prevalence of sarcopenia in Thai adults aged between 60 and 70 years is highly varied, ranging from 16 to 52%, and rates of up to 68% have been reported for adults aged 80 or older [6–10]. This extensive prevalence range in Thailand likely results from disparities in participant demographics, measurement methodologies, and diagnostic criteria among studies. The AWGS 2019 diagnostic criteria for sarcopenia incorporated a reduced threshold for appendicular muscle mass and muscle function (i.e., muscle strength and physical performance). This revision to the criteria improved their diagnostic accuracy for Asian subjects [5]. Only 3 studies have employed the AWGS 2019 diagnostic criteria in the Thai older adult population. However, the studies’ limited sample sizes and region-focused nature mean that they captured only a fragment of the national picture [8–10]. A current and precise understanding of the disease prevalence is crucial for shaping evidence-driven, locale-specific therapeutic and preventive approaches for the Thai older adult population.

The present national epidemiological survey aimed to identify the prevalence of sarcopenia among Thai community-dwelling older adults according to the AWGS 2019 criteria. Additionally, we investigated the prevalence of possible and severe sarcopenia in this population and explored the associated risk factors.

## Methods

This nationwide cross-sectional epidemiological study was conducted from March 2021 to August 2022. Participants were recruited from 12 provinces representing the six primary geographical regions of Thailand. Our recruitment employed a stratified multistage sampling method that was meticulously designed to represent the diverse older Thai population while minimizing sampling errors. Initially, we segregated the sample into six strata, each corresponding to one of Thailand’s major geographic regions, with 500 participants in each stratum. For each region, two provinces were randomly selected. Subsequently, ten enumeration districts covering urban and rural areas were randomly selected within these provinces. We randomly sampled 25 eligible participants from each enumeration district, as depicted in Supplementary Fig. 1. The study included Thai adults aged 60 or older who had resided in the selected households for at least 6 months. We excluded individuals unable to walk independently, those bedridden, or those with neuromuscular disorders or severe comorbidities affecting performance-based test capability. The Central Research Ethics Committee of the National Research Council of Thailand authorized the study protocol (approval number CREC-023/2021).

### Data collection

Eligible participants were invited to the local primary healthcare center for data collection. After obtaining written informed consent, we collected participant demographic and medical history data, including age, sex, body mass index (BMI), Charlson comorbidity index, comorbidities, and current smoking or alcohol consumption status. Bioelectrical impedance analysis (BIA) was conducted during the morning session to maximize test reliability [11]. Participants were instructed to refrain from caffeine or food consumption for 4 h before the morning assessments, with only 1 or 2 glasses of water permitted during that time. Trained research assistants executed all evaluations, and each site had a designated professional quality controller to ensure questionnaire quality and protocol adherence.

### Diagnostic criteria

We employed the AWGS 2019 criteria to define “sarcopenia” as an age-related loss of skeletal muscle mass combined with low muscle strength and/or low physical performance [5]. The AWGS 2019 also introduced the classifications of “possible sarcopenia” (characterized by low muscle strength or poor physical performance) and “severe sarcopenia” (involving abnormalities in muscle mass, strength, and physical performance). Poor physical performance is identified by abnormal gait speed or inferior results in a 5-time chair stand test. Participants demonstrating deficiencies in either of these assessments are classified as having poor physical performance. Participants with normal muscle mass but low muscle strength or poor physical performance were categorized as having “dynapenia [12].”

### BIA

Appendicular skeletal mass (ASM) combines the lean muscle mass from the upper and lower limbs. This study used BIA and a dual-frequency body composition monitor (Tanita RD-545, Tanita Corporation, Tokyo, Japan) to assess ASM. This device is straightforward, noninvasive, cost-effective, and efficient, making it well suited for community-based surveys. The Tanita RD-545 model is reliable and validated for determining ASM in Thai older adults [13]. Participants stood barefoot and with clean feet on metal footpads while extending their arms and grasping the BIA device, per the manufacturer’s guidelines. The AWGS 2019 cutoffs for diminished muscle mass, adjusted by height, are ASM < 7.0 kg/m^2^ for men and ASM < 5.7 kg/m^2^ for women. BIA was also utilized to measure total lean mass, total fat mass, and body fat percentage.

### Handgrip strength

We assessed muscle strength using handgrip strength, as recommended by the AWGS 2019 criteria. Participants were asked to squeeze a digital Smedley spring hand dynamometer (Takei 5401 Digital Dynamometer; Takei, Tokyo, Japan) with maximum effort while standing with fully extended arms at their sides [14]. The grip size was adjusted for each participant’s hand size, and 2 or 3 trials were performed, with the highest output value recorded. Handgrip strengths < 28 kg in males and < 18 kg in females were classified as low muscle strength, per the AWGS 2019 criteria.

### Gait speed

Gait speed measurement is a simple, quick, and convenient test for evaluating physical performance in older adults and has been validated for this purpose [15]. Gait speed was determined over a 5-meter distance using a stopwatch, with participants walking in their customary gait pattern. Participants were instructed to walk from a standing position at their regular pace without deceleration along an 11-meter marked path. They repeated this test twice. The average time to cover the 3- to 8-meter section was recorded, and gait speed was computed. The AWGS 2019 suggests a cutoff value of < 1.0 m/s. The timing was recorded to the nearest 0.01 s.

### Five times sit-to-stand test

The 5 times sit-to-stand test (5TSTS) is widely employed to gauge functional strength in the lower extremities. It is based on the time a participant requires to transition from sitting to standing, and vice versa, five times consecutively. Participants were instructed to sit on a chair with crossed arms against the backrest and then stand and sit as quickly as possible five times. The timing began at the initial sitting position and ended at the final fully seated position after the fifth stand, recorded to the nearest 0.01 s. The 5TSTS is a validated method for evaluating physical performance in older adults [16]. A 5TSTS time of ≥ 12 s was defined as low physical performance per the AWGS 2019 criteria.

### Timed up-and-go test

The timed up-and-go (TUG) test is a performance-based measure of functional mobility and has been validated for older adults [17]. Participants were asked to stand up from an armchair, walk 3 m straight to a floor marker at a typical pace, turn, and reseat themselves. The time taken to complete this action was noted.

### Anthropometric measurements

Anthropometric measurements (weight, height, BMI, and calf circumference) were obtained. Height was measured to the nearest 0.01 cm using a stadiometer, while body weight was measured to the nearest 0.1 kg using a weighing scale. BMI was derived by dividing weight (in kilograms) by the square of height (in meters). For calf circumference, participants stood while the most prominent part of the dominant side’s calf was measured in 0.1 cm increments using a tape measure without tightening. The cutoffs for suboptimal calf circumference were < 33 cm for females and < 34 cm for males per the AWGS 2019 criteria.

### Sample size calculation

The required sample size was determined using a single population proportion formula: N = Z^2^_1−α/2_ x (p x [1–p]/d^2^), where N = required sample size, Z = 95% confidence interval, d = precision, and *p* = prevalence of sarcopenia in older adults. Research by Sri-on et al. [10] revealed that the prevalence of sarcopenia among Thai community-dwelling older adults is 31.6%. Thus, the required sample size for a precision of 0.018 and Z = 1.96 was 2447 participants.

### Statistical analysis

Continuous variables are represented as the means and standard deviations for normally distributed data and as medians and interquartile ranges for nonnormally distributed data. Categorical variables are reported as numbers and percentages. Each metric and outcome measure underwent a normality assessment using the Shapiro–Wilk test. An unpaired t-test was used to compare quantitative variables between sarcopenia and nonsarcopenia participant groups. The chi-square test was used to compare categorical variables between groups.

After the initial analysis, a multivariable logistic regression model evaluated the independent associations between each potential explanatory variable and the condition of sarcopenia. Variables with a univariate significance level of 0.20 or less or those deemed clinically relevant were included in the model. Given the analysis’s explanatory nature, a significance level of *P* < 0.20 was selected as the cutoff for maintaining factors in the final model through a forward stepwise procedure. The adjusted odds ratios (aORs) and their 95% confidence intervals were reported for the final regression model. Analyses were conducted using PASW Statistics, version 18 (SPSS Inc, Chicago, IL, USA). A *P* value of less than 0.05 was considered statistically significant.

## Results

The study commenced with 2543 participants, 87 of whom were excluded due to their inability to maintain a stable stance during the BIA assessment. Thus, subsequent analyses included 2456 (96.4%) community-dwelling individuals of advanced age. Table 1 summarizes their demographic and clinical characteristics. The participants had a mean age of 69.0 years, ranging from 64.0 to 73.0 years, with a majority being female (63.6%). The mean Charlson comorbidity index was 3.0, indicating moderate severity of health status. The mean BMI was 24.2 kg/m^2^.

**Table 1:** Participant demographics and characteristics in the study

Variable	All (*N* = 2456)	AWGS 2019 definition		
		Nonsarcopenia (*n* = 2011)	Sarcopenia (*n* = 445)	*P*
Age, mean ± SD	69.0 ± 6.1	68.5 ± 5.8	71.3 ± 7.0	< 0.001
Age, n (%)				
60–69 y 70–79 y ≥ 80 ys	1444 (58.8%) 841 (34.2%) 171 (7.0%)	1244 (61.9%) 663 (33.0%) 104 (5.1%)	200 (44.9%) 178 (40.0%) 67 (15.1%)	< 0.001
Female, n (%)	1562 (63.6%)	1354 (67.3%)	208 (46.7%)	< 0.001
Body mass index, mean ± SD	24.2 ± 4.3	25.2 ± 3.9	19.6 ± 2.8	< 0.001
Body mass index, kg/m^2,^ n (%)				
< 18.5 18.5–24.9 ≥ 25.0	197 (8.0%) 1279 (52.1%) 980 (39.9%)	37 (1.8%) 1009 (50.2%) 965 (48.0%)	160 (35.9%) 270 (60.7%) 15 (3.4%)	< 0.001
Charlson comorbidity index, mean ± SD	3.0 ± 1.1	3.0 ± 1.1	3.2 ± 1.0	0.002
Charlson comorbidity index, n (%)				
1–2 3–4 ≥ 5	872 (35.5%) 1369 (55.7%) 215 (8.8%)	752 (37.4%) 1090 (54.2%) 169 (8.4%)	120 (27.0%) 279 (62.7%) 46 (10.3%)	< 0.001
Suboptimal calf circumference, n (%)	1203 (49.0%)	792 (39.4%)	411 (92.4%)	< 0.001
Current smoking	305 (12.4%)	199 (9.9%)	106 (23.8%)	< 0.001
Alcohol (≥ 3 units/day)	147 (6.0%)	106 (5.3%)	41 (9.2%)	0.002
Total fat mass (kg)	18.6 ± 8.7	20.2 ± 8.5	11.4 ± 5.8	< 0.001
Body fat (%)	30.7 ± 10.4	32.5 ± 9.8	22.4 ± 9.01	< 0.001
Total lean mass (kg)	37.9 ± 7.1	38.6 ± 7.1	34.9 ± 5.9	< 0.001
ASM (kg)	17.3 ± 4.0	17.9 ± 3.9	14.5 ± 2.9	< 0.001
ASM/height^2^ (kg/m^2^)	7.1 ± 1.2	7.3 ± 1.1	5.8 ± 0.7	< 0.001
Hand-grip strength (kg)	21.9 ± 7.0	22.2 ± 7.0	20.3 ± 6.6	< 0.001
Gait speed (m/s)	1.2 ± 0.3	1.0 ± 0.2	0.9 ± 0.2	0.016
Timed up-and-go test (s)	11.7 ± 3.7	11.5 ± 3.6	12.3 ± 4.0	0.016
Five times sit-to-stand test	16.3 ± 4.8	16.2 ± 4.7	16.7 ± 5.1	0.172
History of falling, n (%)	539 (22.0%)	455 (22.6%)	84 (18.9%)	0.084
Heart failure, n (%)	19 (0.8%)	17 (0.8%)	2 (0.4%)	0.338
Chronic obstructive pulmonary disease, n (%)	51 (2.1%)	30 (1.5%)	21 (4.7%)	< 0.001
Diabetes mellitus, n (%)	439 (17.9%)	382 (19.0%)	57 (12.8%)	0.002
Chronic kidney disease, n (%)	47 (1.9%)	37 (1.8%)	10 (2.2%)	0.570

**Abbreviations**: ASM, appendicular skeletal mass; SD, standard deviation

**Fig. 1:**
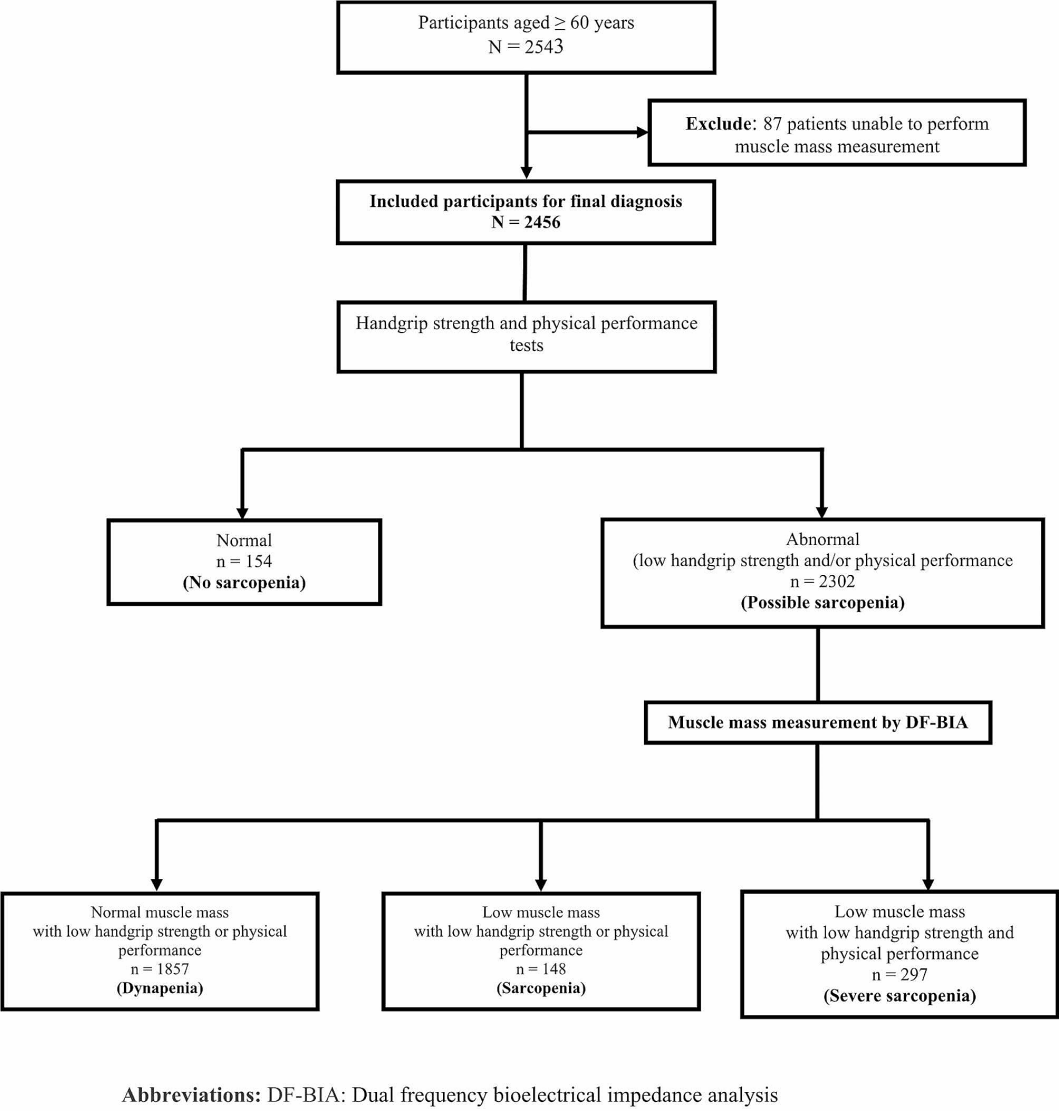
Study design flow diagram

**Fig. 2:**
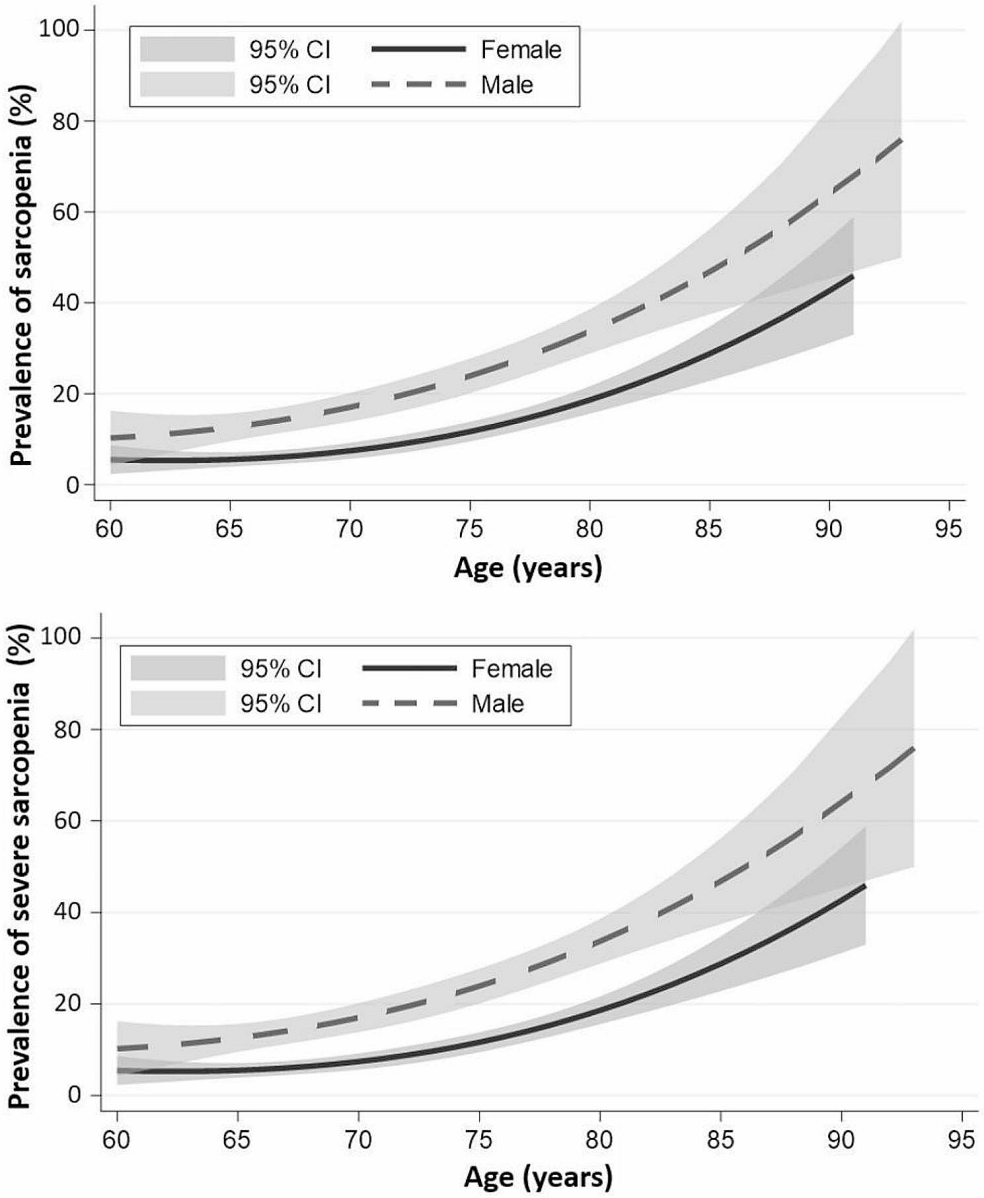
Prevalence of sarcopenia and severe sarcopenia in different age groups for males and females

**Fig. 3:**
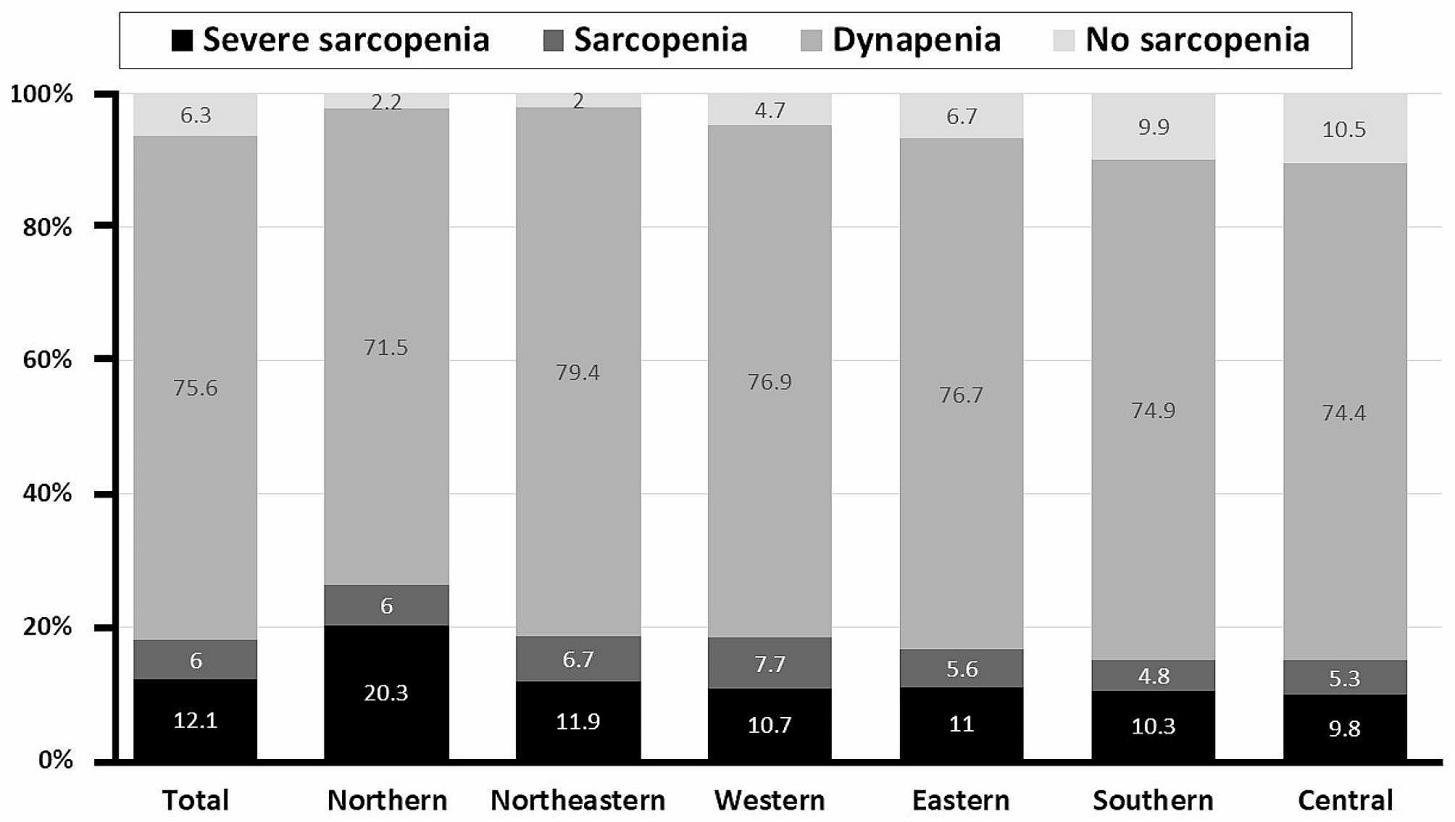
Overall prevalence of dynapenia, sarcopenia, and severe sarcopenia by geographic region in Thailand

Muscle strength and physical performance assessments revealed that only 6.3% of the cohort (154/2456 patients) achieved normal results in both tests. The remaining 93.7% (2302/2456 patients) were diagnosed with possible sarcopenia. Subsequent BIA for muscle mass, using the AWGS 2019 cutoff values, classified 75.6% (95% CI = 73.9–77.3) of the participants (1857/2456 patients) as having dynapenia. This resulted in an overall sarcopenia prevalence of 18.1% (95% CI = 16.7–19.7), comprising 445/2456 patients. Among the sarcopenia-diagnosed participants, 66.7% (297/445 patients) were categorized as severe sarcopenia cases (Fig. 1). Compared to females, males had a higher prevalence of sarcopenia and severe sarcopenia at all ages, with the difference in the male and female age-specific prevalence becoming markedly greater with age progression (Fig. 2). Geographically, the prevalence of sarcopenia was highest in the northern region (26.3%), followed by the northeastern (18.6%), western (18.4%), eastern (16.6%), southern (15.2%), and central regions (15.1%; Fig. 3).

Comparatively, the sarcopenia group had a higher mean age (71.3 vs. 68.5 years, *P* < 0.001), a larger male proportion (53.3% vs. 32.7%, *P* < 0.001), a lower mean BMI (19.6 vs. 25.2, *P* < 0.001), and a slightly higher mean Charlson comorbidity index (3.2 vs. 3.0, *P* = 0.002) than the nonsarcopenia group. Additionally, the sarcopenia group had a significantly higher occurrence of suboptimal calf circumference (92.4% vs. 39.4%, *P* < 0.001). The sarcopenic population had significantly lower mean values for ASM (14.5 kg vs. 17.9 kg, *P* < 0.001), ASM adjusted by height squared (5.8 kg/m^2^ vs. 7.3 kg/m^2^, *P* < 0.001), total fat mass (11.4 kg vs. 20.2 kg, *P* < 0.001), and % body fat (22.4% vs. 32.5%, *P* < 0.001).

Regarding muscle strength and physical performance, sarcopenic participants demonstrated significantly lower mean handgrip strength (20.3 kg vs. 22.2 kg, *P* < 0.001), slower mean gait speed (0.9 m/s vs. 1.0 m/s, *P* = 0.016), and longer TUG test completion time (12.3 s vs. 11.5 s, *P* = 0.016). However, the sarcopenia and nonsarcopenia groups showed no significant difference in their 5TSTS performances. Participants diagnosed with sarcopenia exhibited a higher proportion of chronic obstructive pulmonary disease (COPD), current smoking, and alcohol consumption (≥ 3 units per day) but a lower proportion of diabetes mellitus.

Univariable and multivariable logistic regression analyses were executed to pinpoint the clinical variables significantly linked to sarcopenia (Table 2). The most significant association with sarcopenia was a BMI of less than 18.5 kg/m^2^, with an AOR of 11.7 (95% CI = 7.8–17.4, *P* < 0.001). Other significant clinical risk factors were lower calf circumference (AOR = 6.3, 95% CI = 4.3–9.5, *P* < 0.001), male sex (AOR = 2.8, 95% CI = 2.2–3.7, *P* < 0.001), COPD (AOR = 2.3, 95% CI = 1.1–5.0, *P* = 0.031), advanced age (AOR = 2.1, 95% CI = 1.3–3.3, *P* = 0.001), and longer TUG test completion time (AOR = 1.1, 95% CI = 1.0–1.1, *P* < 0.001).

**Table 2:** Univariate and multivariate of sarcopenia risk factors among Thai community-dwelling older adults

Factors	Univariate			Multivariate*		
	Odds ratio	95% CI	*P*	Odds ratio	95% CI	*P*
Age						
60–69 y 70–79 y ≥ 80 y	1 1.670 4.007	1.336, 2.087 2.848, 5.638	**< 0.001** **< 0.001**	1 1.235 2.096	0.931, 1.639 1.337, 3.285	0.143 **0.001**
Sex (ref, female)	2.348	1.907, 2.892	**< 0.001**	2.829	2.153, 3.716	**< 0.001**
Body mass index						
18.5–24.9 kg/m^2^ < 18.5 kg/m^2^ ≥ 25.0 kg/m^2^	1 16.160 0.058	11.030, 23.676 0.034, 0.098	**< 0.001** **< 0.001**	1 11.660 0.151	7.804, 17.423 0.086, 0.264	**< 0.001** **< 0.001**
Suboptimal calf circumference	18.590	12.957, 26.673	**< 0.001**	6.340	4.247, 9.463	**< 0.001**
Timed up-and-go test (s)	1.048	1.022, 1.075	**< 0.001**	1.077	1.040, 1.116	**< 0.001**
Charlson comorbidity index						
1–2 3–4 ≥ 5	1 1.604 1.706	1.270, 2.025 1.168, 2.491	**< 0.001** **0.006**			
Chronic obstructive pulmonary disease	3.271	1.854, 5.768	**< 0.001**	2.316	1.081, 4.959	**0.031**
Chronic kidney disease	1.226	0.605, 2.485	0.571			
Current smoker	2.847	2.189, 3.702	**< 0.001**			
Alcohol intake of ≥ 3 units/d	1.824	1.252, 2.657	**0.002**			
R^2^ = 0.482						

## Discussion

With its rapidly aging population, Thailand is anticipated to transition into a superaged society in the coming decade. Sarcopenia, an age-related skeletal muscle disease characterized by muscle mass loss, diminished strength, and physical performance, is a significant concern. Notably, this research is among the few to explore sarcopenia prevalence and stands as the first nationwide investigation using the AWGS 2019 criteria for the Thai population. As defined by AWGS 2019, the prevalence of sarcopenia among Thai community-dwelling individuals of advanced age was 18.1%. Furthermore, our study identified age, sex, BMI, calf circumference, prolonged TUG test duration, and a history of COPD as contributing factors to sarcopenia.

The sarcopenia prevalence in our study was notably lower than the 31.6% (95% CI = 28.6–34.7) reported by Sri-on et al., who also employed the AWGS 2019 criteria among Thai community-dwelling older adults in the capital. Their study further differentiated between sarcopenia and severe sarcopenia, with prevalences of 22.2% (95% CI = 25.2–31.2) and 9.4% (95% CI = 7.5–11.5), respectively (Table 3) [10]. In a separate study, Khongsri et al. reported a sarcopenia prevalence of 30.5% (95% CI = 25.0–36.5) among older adults in Thailand’s urban areas [7]. However, our work indicated a lower overall prevalence of sarcopenia (18.1%). We speculate that these discrepancies might be due to differences in population characteristics, habitats, physical activity levels, and incomes. Supporting this, Tuangratanon et al. reported an inverse relationship between physical activity and income. For example, individuals in rural settings, often engaged in agriculture, typically have higher physical activity and energy expenditures but lower incomes than their urban counterparts [18]. Therefore, compared with prior studies focusing on older adults in urban settings, our study suggests that older adults across urban and rural Thailand tend to have a lower prevalence of sarcopenia. Additionally, within Southeast Asia, the prevalence of sarcopenia varies significantly, ranging from 17.6 to 54.3%. This variation is influenced by factors such as study population size and characteristics, demographic profiles, geographical regions, and diagnostic approaches [19].

**Table 3:** Literature review of previous studies reporting the prevalence and risk factors of sarcopenia among older Thai adults according to the AWGS 2019 criteria

Paper	Publication year	Study type	Study population	Sample size	Sarcopenia prevalence	Associated risk factors
Sri-on et al. [10]	2022	Cross-sectional	Older adults aged > 60 years in Bangkok	892	31.6%	Age ≥ 70 years, low BMI, and inadequate nutrition
Yuenyongchaiwat et al. [9]	2022	Prospective cohort	Older adults aged ≥ 60 years in community-dwelling	205	21.5%	Low physical activity
Therakomen et al. [8]	2020	Cross-sectional	Older adults aged > 60 years in outpatient clinic at King Chulalongkorn Memorial Hospital	330	10%	Age ≥ 70 years, prefrailty, lower physical activity
The present study		Cross-sectional, nationwide	Older adults aged ≥ 60 years in community-dwelling	2456	18.1%	Age ≥ 80 years, male sex, low BMI, lower leg calf circumference, increased time in TUG test, and history of COPD

**Abbreviations**: BMI, body mass index; COPD, chronic obstructive pulmonary disease; TUG, timed up-and-go

The current investigation observed a high prevalence of dynapenia, an age-related loss of muscle strength without a concomitant reduction in muscle mass. This phenomenon can be attributed to two primary causes. Dynapenia may either represent the initial manifestation of sarcopenia or be linked to other factors, such as neural activation impairments or a decline in the intrinsic force-producing capability of skeletal muscle [20]. Earlier research has shown that individuals with dynapenia exhibit higher morbidity and mortality rates than sarcopenia patients [20, 21]. Identifying such individuals for early intervention is crucial to preempt sarcopenia onset and address underlying neural or muscular dysfunctions.

Previous studies have identified numerous risk factors that contribute to the development of sarcopenia: advanced age, sex, physical inactivity, history of falls, underweight status, malnutrition, cognitive impairment, and existing illnesses such as diabetes and respiratory diseases [8–10, 22]. It is important to interpret these factors cautiously due to potential reverse causality and confounding that might impact the results, and they may not be generalizable to all older adult populations. Our multivariable logistic regression analysis revealed significant associations between sarcopenia and advanced age, male sex, low BMI, calf circumferences below 34 cm in males or 33 cm in females, COPD, and elongated TUG test durations.

Advancing age is associated with sarcopenia due to reduced growth hormone production and elevated serum inflammatory markers, resulting in an accelerated loss of muscle mass after the age of 75 years [23, 24]. Our observations indicate that males are twice as likely to develop sarcopenia as females, a finding corroborated by prior research. This increased susceptibility in males may stem from diminished free testosterone levels, suboptimal physical activity, a higher prevalence of cardiovascular disease, and suboptimal insulin-like growth factor-1 levels [25].

A positive correlation between muscle indicators and BMI and body weight was evident in the older adult demographic. A diminished BMI is a recognized sarcopenia predictor, potentially yielding biomarkers such as decreased triglycerides [26]. Consistent with our observations, numerous investigations have established a positive correlation between calf circumference and muscle mass irrespective of obesity status or age [27, 28]. Resistance exercise programs in adulthood and early elderly should be developed to maintain muscle wasting and weakness.

The TUG test serves as a practical indicator reflecting sarcopenia risk. Despite its utility, further research is needed to establish a definitive cutoff for sarcopenia assessment using this metric. COPD and sarcopenia share similarities and possible interactions, including common risk factors (e.g., advanced age, low BMI, physical inactivity, malnutrition), suggesting a shared biology. This possibility is supported by extensive evidence [29, 30]. Chronic systemic inflammation is a pivotal component of the pathogenesis and progression of COPD and sarcopenia. However, other factors, such as insufficient energy intake, oxidative stress, and reduced muscle blood flow, may also be involved [30, 31].

The primary strength of this study is its status as the first large-scale investigation of sarcopenia prevalence and associated risk factors in the Thai community-dwelling older adult population at a national level. This research comprehensively sampled urban and rural locales across Thailand’s 6 geographically diverse regions using the AWGS 2019 criteria. The study identified BMI, calf circumference, and the TUG test as invaluable metrics for effective sarcopenia screening among community-dwelling older adults. However, certain constraints must also be acknowledged. The cross-sectional design may limit our ability to infer causative relationships between risk elements and sarcopenia. Moreover, specific risk parameters, such as BMI or disease severity, could evolve, necessitating prolonged observation for conclusive validation.

## Conclusions

Our results indicate that advancing age, male sex, suboptimal BMI, suboptimal calf circumference, elongated TUG test durations, and underlying COPD elevate the risk of developing sarcopenia. A holistic approach encompassing multifaceted lifestyle modifications and comprehensive nutritional backing throughout adulthood is imperative to sustain muscle vigor and physical strength and, in turn, mitigate sarcopenia risk. National health strategies should prioritize these interventions, with an emphasis on males.

The regions and corresponding provinces were the northern (Chiang Mai, Lampang), northeastern (Sri Saket, Udon Thani), western (Prachuap Khiri Khan, Kanchanaburi), eastern (Sa Kaeo and Rayong), southern (Songkhla and Nakhon Si Thammarat), and central (Phitsanulok, Nakhon Sawan) regions.

### Electronic supplementary material

Below is the link to the electronic supplementary material.

**Supplementary Material 1**: **Supplementary Fig. 1**. The flowchart demonstrates the multistage stratified random sampling process

## Data Availability

The data supporting this study’s findings are available from the corresponding author upon reasonable request. The data are not publicly available due to privacy or ethical restrictions.

## Abbreviations

AWGS: Asian Working Group for Sarcopenia
BMI: Body mass index
BIA: Bioelectrical impedance analysis
ASM: Appendicular skeletal mass
5TSTS: 5 times sit-to-stand test
TUG: Timed up-and-go test
COPD: Chronic obstructive pulmonary disease
