# Supplementary material for: Sarcopenia in Thai community-dwelling older adults: a national, cross-sectional, epidemiological study of prevalence and risk factors
Source: BMC Public Health. 2024 Jan 27;24:311. doi: 10.1186/s12889-024-17804-7 (PMC10821311; doi:10.1186/s12889-024-17804-7)

**Supplementary Figure 1.** The flowchart demonstrates the multistage stratified random sampling process


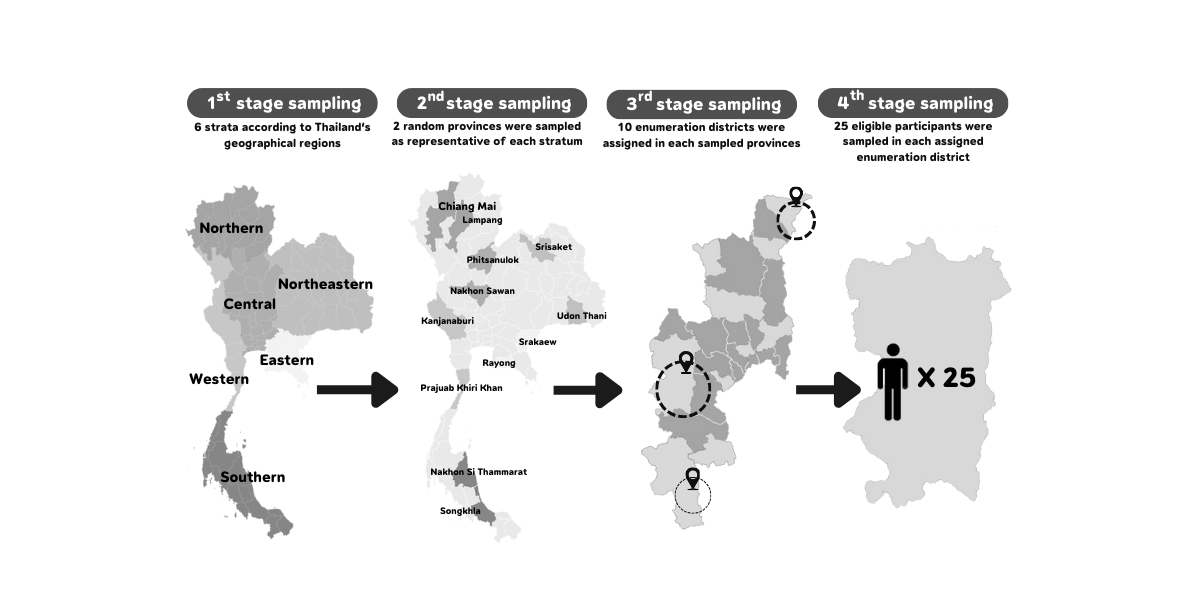

Supplement: Supplementary file 1 — Supplementary Material 1: Supplementary Fig. 1. The flowchart demonstrates the multistage stratified random sampling process [file 12889_2024_17804_MOESM1_ESM.docx]
